# The Bayesian lens and Bayesian blinkers

**DOI:** 10.1098/rsta.2022.0144

**Published:** 2023-05-15

**Authors:** Matthew Stephens

**Affiliations:** Department of Statistics and Department of Human Genetics, University of Chicago, Chicago, IL, USA

**Keywords:** Bayesian, frequentist, confidence interval, *p*-value, maximum likelihood

## Abstract

I discuss the benefits of looking through the ‘Bayesian lens’ (seeking a Bayesian interpretation of ostensibly non-Bayesian methods), and the dangers of wearing ‘Bayesian blinkers’ (eschewing non-Bayesian methods as a matter of philosophical principle). I hope that the ideas may be useful to scientists trying to understand widely used statistical methods (including confidence intervals and p-values), as well as teachers of statistics and practitioners who wish to avoid the mistake of overemphasizing philosophy at the expense of practical matters.

This article is part of the theme issue ‘Bayesian inference: challenges, perspectives, and prospects’.

## 1. Introduction

I spend much of my time as a statistician developing, applying and teaching Bayesian statistical methods. I do not remember a defining moment when I ‘became a Bayesian’, but during my early statistical education I do recall being uneasy about some aspects of the prevailing frequentist philosophy, and a general feeling of ‘that makes so much sense!’ when I later learned more about the Bayesian approach. I was lucky to be part of a generation where Bayesian statistics was, pretty much, mainstream—something that Prof. Adrian Smith, in honour of whom this piece was commissioned, helped bring about with contributions such as [1,2]. As a result, and perhaps unlike earlier practitioners, I have felt little pressure to defend or apologize for taking a Bayesian approach. However, the widespread use of frequentist (and other non-Bayesian) methods in practice has certainly cast me, at times, in the role of Evangelist, trying to persuade people to drop their frequentist methods in favour of Bayesian alternatives. And as a teacher, I have the job of presenting statistical tools that are widely used, and yet, philosophically, make no sense (I am looking at you, confidence intervals). The Bayesian lens and Bayesian blinkers are concepts I have formulated as I have learned to navigate these waters.

## 2. The Bayesian lens

Many statistical procedures in common use are derived or motivated by non-Bayesian arguments. However, many of these procedures have a Bayesian interpretation if one looks for it. I call finding this interpretation ‘looking through the Bayesian lens’. Looking at a procedure through the Bayesian lens usually means working out what prior assumptions—and, perhaps, loss function—a particular procedure corresponds to. The idea is that this can give additional insights into the procedure, highlighting implicit assumptions that are being made, or suggesting when the procedure might be expected to perform well, and, conversely, when it might be better avoided. And, when trying to persuade someone to adopt a new Bayesian procedure, it can be helpful to cast existing procedures in a Bayesian way, to better highlight key differences.

Here, I illustrate this idea with some simple examples. Neither the examples nor the idea itself are new; indeed [3] has the same idea in mind when he describes the potential for a ‘Bayesian lamp’ to shine light on non-Bayesian methods, and Prof. Smith himself has made relevant contributions by connecting Bayes Factors with model choice criterion such as AIC [4]. However, I hope that putting these examples together in a single place has some value, and perhaps helps bring new attention to them.

### (a) Confidence intervals

There is, perhaps, no commonly used statistical procedure that is harder to explain than a confidence interval. To remind readers, a 95% confidence interval for a parameter θ is a random interval, [A(X),B(X)], depending on the data X, constructed in such a way that the interval has 95% probability of containing θ

2.1 $$P_{X|\theta }(A(X)\leq \theta \leq B(X))=0.95,$$

where the subscript on P emphasizes that the probability is over the distribution p(X|θ).

This is all well and good, but what does it mean if I report, for example, ‘the interval [0.1,0.9] is a 95% confidence interval for θ?’ This report seems to suggest that I am ‘confident’ that θ lies in the range 0.1–0.9. How confident? Well, presumably 95% confident. However, as statistics professors around the world try (and largely fail) to teach our students, this does not mean that there is a 95% *probability* that θ lies in the range 0.1–0.9, because in the definition of a confidence interval θ is fixed and it is the interval that is random. Once the mathematical expression for the interval A(X),B(X) has been replaced with *actual numbers* the meaning of the confidence interval becomes, in my opinion, impossible to describe. (The situation is much worse than with p-values: I can tell you a reasonably simple question to which the p-value is the answer, but not so for confidence intervals.)

The consequence of this difficulty is, I claim, that almost every *user* (as opposed to theorist) of statistics around the world misinterprets the phrase ‘the interval [0.1,0.9] is a 95% confidence interval for θ?’ as meaning there is a 95% probability that θ lies in the interval [0.1,0.9]. That is, they misinterpret the confidence interval as a Bayesian interval, often referred to as a Bayesian ‘credible interval’.

In ‘Subjective Probability and Statistical Practice’ [5], §4, Savage looks at confidence intervals through the Bayesian lens. Specifically, he considers the case of the standard normal confidence interval, which is probably the most commonly encountered confidence interval in practice. His example involves the weighing of a potato, and is worth reading for its amusement value alone. Here, I paraphrase and generalize his analysis to the situation where we have a measurement $X∼N(\theta ,s^{2})$ for some known standard error s, which leads to the familiar 95% confidence interval for θ of X±1.96s. Savage considers a Bayesian analysis in which the prior distribution varies ‘at most a few per cent’ in the interval [X−5s,X+5s], and is never enormously larger outside this interval than inside the interval. For brevity, I will simply call a prior satisfying these assumptions ‘diffuse’. For such a diffuse prior, the posterior distribution for θ is approximately $\theta |X∼N(X,s^{2})$, and so in particular

2.2 $$P_{\theta |X}(\theta \in [X-1.96s,X+1.96s]|X)\approx 0.95,$$

where here the probability statement is over θ. Putting some numbers in to make this point more concrete, let X=0.5 and s=0.2 then the standard 95% confidence interval for θ is approximately [0.1,0.9], and $P_{\theta |X}(\theta \in [0.1,0.9]|X=0.5)\approx 0.95$. Savage concludes:

Though this is much the kind of conclusion that is usually ridiculed in the statistics classroom, I hope you now feel that, in the presence of reasonable assumptions about your own initial subjective probability, it is not ridiculous but true.

In other words, looking at the standard (normal) 95% confidence interval through a Bayesian lens, we see that it is also a 95% credible interval under a diffuse prior. Thus we can usefully recast a ‘mistake’—misinterpreting a confidence interval as a credible interval—as an ‘assumption’, that the prior is diffuse. This recasting suggests that the misinterpretation is only problematic if there is reason to believe that the prior is not diffuse.

In what settings might belief in a diffuse prior be questionable? One answer to this might be: any setting where there is reason to believe, *a priori*, that θ is likely very close to 0—perhaps in settings where one might be tempted to test the null hypothesis $H_{0}:\theta =0$.^1^ In such settings misinterpreting the confidence interval as a credible interval is problematic because it corresponds to assuming that the prior is diffuse. And since almost every user of statistics misinterprets confidence intervals in this way, using a standard confidence interval in such settings seems, to me, problematic and something to be avoided.^2^ While I appreciate the general benefits of focusing on estimation rather than, or in addition to, testing, it seems curious to me that confidence intervals have largely avoided the recent scrutiny experienced by p-values and the routine use of 0.05 as a significance threshold [6–8]; see [9] for relevant discussion.

Although we focused our Bayesian lens here on the (widely used) normal confidence interval, a similar result applies for any location family of distributions. That is, suppose X has density depending on a location parameter θ, $f_{X}(x|\theta )=\phi (x-\theta )$ for some probability density function ϕ, with corresponding cumulative distribution function (cdf) Φ. Then the usual 95% confidence interval, $\left[X+\Phi^{-1}(0.025),X+\Phi^{-1}(0.975)\right]$ is, approximately, also a Bayesian credible interval under a diffuse prior. It is possible that similar ideas apply, at least approximately, to other widely used confidence intervals, although not all confidence intervals have a Bayesian interpretation. My own view is that, philosophically, confidence intervals make no sense; see [10] pp. 24–25 for an example that I believe illustrates their fundamental philosophical flaw. However, the Bayesian lens rescues the standard normal confidence interval as a practical tool, and may help explain why these confidence intervals remain widely used in practice despite philosophical problems.

### (b) *p*-values

We move now to that other widely used tool of frequentist inference, the p-value. There is, of course, a close connection between p-values and confidence intervals: for example, for the normal observation $X∼N(\theta ,s^{2})$ considered above, the standard 95% confidence interval contains 0 if and only if the usual (two-sided) p-value testing $H_{0}:\theta =0$ satisfies p<0.05. Thus I begin by translating the Bayesian lens results above to apply to p-values.

As noted above, under Savage’s ‘diffuse’ prior assumptions, the posterior distribution for θ is approximately $\theta |X∼N(X,s^{2})$. If X>0 then the one-sided p-value testing $H_{0}:\theta \leq 0$ versus θ>0 is 1−Φ(X/s), where Φ is the cdf of the standard normal distribution. This is also the posterior probability that θ>0

2.3 $$p\;\text{(one-sided) }=1-\Phi \left(\frac{X}{s}\right)=P_{\theta |X}(\theta >0|X).$$

Thus, the one-sided p-value has the following Bayesian interpretation: it is the posterior probability, under a diffuse prior, that the sign of θ is different from the observed sign of X. That is, it is the local false sign rate (lfsr), as defined in [11]. This observation is also made in [3] and is related to Theorem 3.2 in [12], which shows that the infinum of the lfsr over unimodal priors that are symmetric about 0 is p; in essence a diffuse prior achieves this infinum.

In summary, under Savage’s diffuse prior assumptions, the p-value is indeed a *direct Bayesian measure of error probability*, specifically the local false sign rate. This result may seem to be in contrast with the usual message that one should avoid, at all costs, misinterpreting p-values as direct error probabilities. However, it is worth emphasizing that p-values are almost always used in hypothesis testing situations, where there is (presumably) reason to believe that the null, or something close to it, might be true, and so the diffuse prior assumptions typically do not hold. That is, a p-value does correspond to a direct Bayesian measure of error probability, but, somewhat ironically, only under *a prior* that would usually be considered inappropriate for hypothesis testing! See [13] for connections between p-values and Bayesian measures of evidence under priors more appropriate for hypothesis testing settings.

#### (i) Multiple testing and the ‘*p*-value prior’

I now consider the ‘multiple testing’ setting, where a large number of null hypotheses are to be tested. Again, to keep things simple, I will consider the case of normal data. Specifically, consider a series of independent observations

2.4 $$X_{j}|\theta_{j},s_{j}∼N(\theta_{j},s_{j}^{2})\;j=1,\ldots ,p,$$

where the standard deviations $s_{j}$ are fixed and known, and where the goal is to test the null hypotheses, $H_{j}:\theta_{j}=0$. A standard (non-Bayesian) approach would be to compute the z-scores for each test, $z_{j}:=X_{j}/s_{j}$, and a corresponding p-value, $p_{j}$, and rank the tests by their ‘significance’: small p-values being the most ‘significant’. One might use a false discovery rate-based procedure, such as Benjamini–Hochberg [14] or q-value [15] to decide on an appropriate threshold, γ say, and reject all $H_{j}$ for which $p_{j}<\gamma$.

Now, following Wakefield [16], we examine this approach under the Bayesian lens. More specifically, we examine the step of *using the p-values to order the hypotheses* when assessing the strength of evidence against the null. Under what prior assumptions does it make sense to consider that hypotheses with smaller $p_{j}$ are more likely to be false than hypotheses with larger $p_{j}.$^3^ The answer to this question, summarized here, was provided by Wakefield [16].

From a Bayesian perspective, with some mild symmetry assumptions on the loss function, the right way to order the hypotheses is by their posterior probability of being false; that is, by $P(\neg H_{j}|X_{j})$, where $\neg H_{j}$ means ‘not $H_{j}$’, i.e. $\theta_{j}\neq 0$. Assuming all null hypotheses are equally plausible *a priori*, ordering by these posterior probabilities is equivalent to ordering by the Bayes Factors ${\text{BF}}_{j}$, which are given by

2.5 $${\text{BF}}_{j}:=\frac{p(X_{j}|\neg H_{j})}{p(X_{j}|H_{j})}.$$

The numerator in this Bayes Factor is

2.6 $$p(X_{j}|\neg H_{j})=\int p(X_{j}|\theta_{j},s_{j})p(\theta_{j}|s_{j},\neg H_{j})\;d\theta_{j},$$

and computing this requires specification of *a prior* distribution for $\theta_{j}$ under the alternative hypothesis $\neg H_{j}$. [16] assumes $\theta_{j}|s_{j},\neg H_{j}∼N(0,W_{j})$ for some variance $W_{j}$, which gives a nice closed-form expression for the Bayes Factor

2.7 $${\text{BF}}_{j}=\sqrt{\frac{s_{j}^{2}}{s_{j}^{2}+W_{j}}}\exp\left(-0.5z_{j}^{2}\frac{W_{j}}{s_{j}^{2}+W_{j}}\right).$$

Examining this we see that if $W_{j}=cs_{j}^{2}$ for some constant c then *ordering the hypotheses by ${\text{BF}}_{j}$ is the same as ordering the hypotheses by $z_{j}^{2}$* (which is the same as ordering them by the p-values $p_{j}$). On the other hand if, for example, $W_{j}$ is the same for all j, then the ordering obtained using ${\text{BF}}_{j}$ will in general be different than the ordering obtained using $p_{j}$.

Although [16] considered a normal prior for $\theta_{j}$ the analysis is not hard to extend to more general priors. Specifically, the result (ordering by BF = ordering by p-values) requires only that the prior on $\theta_{j}$ scales with $s_{j}$, or in other words $\theta_{j}=s_{j}\mu_{j}$, where $\mu_{j}∼g()$ for any prior g. Following Wakefield, I will call *a prior* of this form a ‘p-value prior’.

Given the centrality of p-values in the way that statistics is usually taught (and, often, applied), ordering hypotheses by their p-values seems so natural that most users of statistics would probably do it without even thinking about it. However, as this analysis shows, ordering hypotheses by their p-values corresponds to making certain implicit assumptions about the non-zero effect sizes $\theta_{j}$, and specifically that the tests j with larger standard deviations $s_{j}$ tend to have larger effect sizes (proportional to $s_{j}$). As Wakefield put it,

⋯ the description of a specific prior that gives identical rankings between Bayes factors and *p*-values [provides] a link between the two approaches, and [allows] the implications of the use of *p*-values to be more easily understood.

Having made this connection, it seems natural to ask when might the ‘p-value prior’ be reasonable? Wakefield conducted his work in the context of genome-wide association studies, where each test is a test for association between a genetic variant (SNP) and some trait of interest (e.g. cholesterol level). In this setting the standard errors $s_{j}$ vary with j in part because the variation of SNPs in the population varies. Indeed, if all SNPs are fully typed in a sample of size n then $s_{j}\approx 1/\sqrt{nf_{j}(1-f_{j})}$, where $f_{j}$ is the ‘minor allele frequency’ (the frequency of the less common of the two alleles, or genetic types) at SNP j. Since n is a constant across j, here the p-value prior assumes that *SNPs with smaller $f_{j}$ tend, on average, to have larger effect sizes $|\theta_{j}|$*. As it happens this assumption is qualitatively plausible, for example because selection may tend to keep variants with large effects at lower frequency. It also has some empirical support [17]. On the other hand, if the SNPs are typed in samples of different size, so $s_{j}\approx 1/\sqrt{n_{j}f_{j}(1-f_{j})}$ where $n_{j}$ varies with j, the p-value prior becomes less attractive. In GWAS analysis, genotype imputation is often used to ‘fill-in’ unobserved genotypes [18], and then the standard error for SNP j also reflects how hard SNP j is to impute. In this case, the p-value prior corresponds to assuming that harder-to-impute SNPs have larger effect sizes, which is unlikely to be true [19].

One can similarly apply the Bayesian lens to more complex testing situations, involving meta-analysis [20] and multivariate tests [21]. The results become more involved, but interesting nonetheless. For example, consider the multivariate (r-variate) normal setting

2.8 $$X_{j}|\theta_{j}∼N_{r}(\theta_{j},V),$$

for some r×r covariance matrix V, and where we wish to test $\theta_{j}=0$. One common way to do this is to use a likelihood ratio test (LRT) statistic. Looking at this test under the Bayesian lens, it can be shown that the LRT is monotonic with the Bayes Factor under the prior $H_{1}:\theta_{j}∼N_{r}(0,\lambda V)$ for any scalar λ. That is, the LRT can be thought of as implicitly assuming that the prior correlation of the effects is the same as the correlation of the errors ($X_{j}-\theta_{j}$). See [21] for related examples and discussion.

Again, for those interested more generally in translating p-values to Bayes Factors, I highly recommend [13]. Other relevant work includes I. J. Good’s Bayes/non-Bayes compromise [22], work on characterizing the distribution of Bayes Factors under the null [23] and recent work on ‘e-values’ [24].

### (c) Estimation

Finally, I turn the Bayesian lens on estimation. Obtaining point estimates in the Bayesian paradigm involves specifying two elements, which are helpful to distinguish: the prior distribution, which captures uncertainty in the parameters to be estimated before seeing the data, and the loss function, which captures how ‘bad’ different amounts of error are considered to be. Once the prior and loss function are specified, the Bayesian simply computes the optimal estimate under that prior and loss function. Thus, looking at an estimation procedure through the Bayesian lens involves saying what prior and loss function a particular estimation procedure corresponds to.

#### (i) Normal means model and James–Stein shrinkage

James & Stein [25] rocked the statistical world with a simple but counterintuitive result on estimation. In brief, they considered the so-called ‘normal means model’

2.9 $$X_{j}|\theta_{j}∼N(\theta_{j},1)\;j=1,\ldots ,p,$$

and showed that the estimator

2.10 $${\hat{\theta }}_{j}^{JS}:=(\frac{1-(p-2)}{\sum_{j}X_{j}^{2}})X_{j},$$

is better than the maximum-likelihood estimate ${\hat{\theta }}_{j}^{mle}=X_{j}$, in that it has smaller expected squared error.

In an influential paper, [26] examined the James–Stein estimator through the Bayesian lens. They noted that under the prior distribution

2.11 $$\theta_{j}∼N(0,A),$$

the posterior distribution for $\theta_{j}$ is

2.12 $$\theta_{j}|X_{j}∼N((1-B)X_{j},1-B),$$

where B=1/(A+1). Thus the James–Stein estimator is the posterior mean when $B=(p-2)/\sum_{j}X_{j}^{2}$, or equivalently $A=[1/(p-2)]\sum_{j}X_{j}^{2}-1$.

Since this value of A depends on the data $x_{1},\ldots ,x_{p}$, this provides a view of the James–Stein estimator as the posterior mean under *a prior* distribution (2.11) where A is *estimated from the data*. That is, it provides an ‘empirical Bayes’ (EB) view of the James–Stein estimator. Since the prior (2.11) requires A≥0, this EB view suggests modifying the estimate for A to impose this constraint. That is, $A={\left([1/(p-2)]\sum_{j}X_{j}^{2}-1\right)}_{+}$ where $z_{+}$ denotes max(0,z). The result is the so-called ‘positive part James–Stein estimator’,

2.13 $${\hat{\theta }}_{j}^{JS+}:={\left(\frac{1-(p-2)}{\sum_{j}X_{j}^{2}}\right)}_{+}X_{j}.$$

This (empirical) Bayesian view suggests that ${\hat{\theta }}_{j}^{JS+}$ will perform well in settings where it is reasonable to assume that the true $\theta_{j}$ are normally distributed, as in (2.11). On the other hand, it also suggests that one can do better in settings where this is not the case. See [11] for examples of more flexible EB approaches to this problem.

#### (ii) Ridge regression

Consider now the multiple regression model

2.14 $$y|\beta ∼N_{n}(X\beta ,\sigma^{2}I_{n}),$$

where $I_{n}$ denotes the n×n identity matrix. Here, I have switched to use the standard statistical notation for regression, so y denotes a vector of observed outcome data on n individuals, X represents an n×p ‘design matrix’ containing the values of some set of covariates or predictor variables, and β is a p-vector of parameters, known as the regression coefficients, which are to be estimated.

The log-likelihood for this regression model (up to an additive constant) is

2.15 $$l(\beta )=-\left[\frac{1}{2\sigma^{2}}\right]{\left(y-X\beta \right)}^{\prime }(y-X\beta ).$$

And, assuming $\left(X^{\prime }X\right)$ is invertible, the maximum-likelihood estimate for β (also known as the ordinary least-squares estimate) is

2.16 $${\hat{\beta }}^{mle}={\left(X^{\prime }X\right)}^{-1}X^{\prime }y.$$

An obvious problem with (2.16) is that it does not exist if $X^{\prime }X$ is not invertible. Furthermore, even if $X^{\prime }X$ is invertible, the maximum-likelihood estimate can be unstable (i.e. have high variance, resulting in low accuracy). According to [27], Hoerl suggested the following ridge regression estimate (in a 1962 article that I have been unable to locate) to address this problem, specifically to ‘control the inflation and general instability of the least-squares estimates’

2.17 $${\hat{\beta }}^{\text{ridge}}={\left(X^{\prime }X+kI\right)}^{-1}X^{\prime }y.$$

[27] characterize ${\hat{\beta }}^{\text{ridge}}$ as the solution to $\max_{\beta }F(\beta )$ where

2.18 $$F(\beta )=-l(\beta )+k||\beta |{|}_{2}^{2},$$

where $||\beta |{|}_{2}^{2}=\sum_{j=1}^{p}\beta_{j}^{2}$ is the squared $l^{2}$ norm. Since F has the form of a log-likelihood plus an additional term that penalizes large values of $\beta_{j}$, this characterization provides a ‘penalized log-likelihood’ interpretation of ridge regression. However, citing earlier work by [28,29] they also note

⋯each ridge estimate can be considered as the posterior mean based on giving the regression coefficients, β, *a prior* normal distribution with mean zero and variance-covariance matrix $\Sigma =\delta_{ij}[\sigma^{2}]/k$.

That is, under prior

2.19 $$\beta ∼N_{p}\left(0,\left(\frac{\sigma^{2}}{k}\right)I_{p}\right),$$

$\beta^{\text{ridge}}=E(\beta |y,X)$. That is, the ridge estimate is the posterior mean under the prior (2.19).

In this quote, Hoerl & Kennard [27] are looking at the ridge regression estimates through the Bayesian lens.^4^ So what do we gain from the Bayesian lens? First, we immediately learn conditions under which estimates of the form (2.17) are optimal. Since the posterior mean is optimal under squared error loss, the ridge estimate is optimal, under squared error loss, when the $\beta_{j}$ are drawn from the normal distribution (2.19). (Actually, the ridge estimate is also the posterior median and the posterior mode, so it is also optimal under other measures of loss.) What we do not immediately know, of course, is how robust the method will be to deviations from this assumption. Nonetheless, it is a start. Arguably it also suggests a way to select a value for k: since the ridge estimate (2.17) is optimal under the model (2.14)–(2.19) it seems natural to use the same modelling assumptions to select k, for example using an EB approach (i.e. by maximizing the marginal likelihood).

#### (iii) Lasso regression

The lasso regression estimate [30] is obtained by replacing the squared $l^{2}$ penalty of ridge regression in (2.18) with an $l^{1}$ penalty, $|\beta {|}_{1}=\sum_{j=1}^{p}|\beta_{j}|$

2.20 $${\hat{\beta }}^{\text{lasso}}=\arg\min-l(\beta )+k|\beta {|}_{1}.$$

It is common to describe $\beta^{\text{lasso}}$ as the Bayesian ‘maximum *a posteriori*’ (MAP) estimate for β under a Laplace prior (also known as double-exponential prior), a fact that is straightforward to verify. This description is, in essence, an examination of lasso through a Bayesian lens.

However, some cautionary remarks are in order. First, and most importantly, the MAP estimate is ultimately not a very ‘Bayesian’ quantity, at least not for continuous parameters. Why is this? Well, the MAP estimate is the optimal estimate under 0–1 loss, which is not a natural loss function for continuous parameters. By contrast, the posterior mean estimate is optimal under squared loss, which is much more natural. Second, lasso is often applied in settings where it is believed that many of the regression coefficients are equal to (or very close to) 0; the Laplace prior does not really seem to capture this belief. Thus one might, perhaps uncharitably, characterize the lasso estimate as corresponding to the Bayes estimate under an inappropriate prior and an inappropriate loss function.

These remarks might suggest asking a different question: ‘Under what prior is the lasso estimate the posterior mean’? I believe the answer to this is that no such prior exists. Because of this I tend to think of the lasso as not really corresponding to a Bayesian procedure, at least not one that I would defend. This does not imply that lasso is not useful in practice, but it does make it harder to use Bayesian arguments to deduce under what situations it might be expected to perform well. Of course, there is extensive frequentist theory to help guide us here; [31] provides a brief review and some numerical studies.

## 3. Bayesian blinkers

Historically, debates over the relative merits of Bayesian versus non-Bayesian methods have often been heated. While I like to think that we have gotten over the worst of this, there remain, undoubtedly, some statisticians with entrenched positions. Furthermore, the fact of the matter is that we all come to a problem with our own set of tools that we feel comfortable with. Thus, in some cases Bayesian methods may be used either because of philosophical intransigence, or simply lack of knowledge or due consideration of possible alternatives. (And, of course, the same applies to non-Bayesian methods.)

I myself have made extensive use of Bayesian methods, and most of my work has a Bayesian component. However, I have learned from experience that not all problems need to be tackled in a Bayesian way. In particular, it is sometimes worth compromising between statistical principle and computational practice. In this section, I discuss two examples where, perhaps, my ‘Bayesian blinkers’ lead me to ignore other possible approaches. Certainly, they are examples where I tackled a problem in a Bayesian way, but now typically use non-Bayesian approaches for these problems.^5^

### (a) Population structure and clustering

The first example is my work on so-called ‘admixture models’ to infer population structure from genetic data [32]. In brief, population structure refers to the fact that some individuals are more genetically similar than others. Thus, a typical first attempt to investigate population structure might involve applying some kind of clustering method to the genetic data. The main contribution of [32] was to extend a standard model-based cluster model to allow that each individual could have *partial membership in each cluster*; that is, in the language of population genetics, to allow that individuals may be ‘admixed’. In essence, we did this by replacing the usual ‘latent variable’ formulation of clustering, in which each individual i is assumed to belong to some latent cluster $z_{i}\in \{1,\ldots ,K\}$, with an assumption that each individual has a latent vector $q_{i}:=(q_{i1},\ldots ,q_{iK})$, where $q_{ik}$ denotes the proportion of membership of individual i in cluster k. In the genetic context, the clusters can be interpreted as ‘ancestral populations’ and $q_{ik}$ is the proportion of individual i’s genome that was inherited from ancestral population k.

We tackled this problem in a Bayesian way, by specifying prior distributions on the unknown parameters—including a Dirichlet prior distribution for each $q_{i}$, i=1,…,n, and prior distributions for the population allele frequencies (cluster means), collectively denoted P—and using Markov Chain Monte Carlo to sample from the joint posterior distributions for $q_{1},\ldots ,q_{n};P$. One can then, of course, use the MCMC samples to obtain both point estimates and measures of uncertainty for these parameters (paying appropriate attention to label-switching [33]). This method became widely used to investigate structure in genetic data, ranging from bacteria like *Helicobacter pylori* [34] through snails [35] and dogs [36] to humans [37]. However, in practice, almost every application of this method focuses entirely on the point estimates for $q_{1},\ldots ,q_{n}$.

Motivated by a desire to speed up inference, [38] introduced faster methods for obtaining point estimates for $q_{1},\ldots ,q_{n}$ based on maximizing the likelihood rather than performing MCMC to sample from the posterior. According to [38] the resulting methods ‘solv[e] problems in minutes that take [the MCMC-based method] hours’. For large datasets I would certainly use this maximum-likelihood method myself to find point estimates, rather than bothering with MCMC. From where I am sitting now this approach seems entirely natural, if not blindingly obvious. And yet the idea of performing maximum-likelihood estimation never occurred to me when we first tackled the problem. Perhaps my Bayesian blinkers were getting in the way.

As a side note, I find it interesting that in related work on ‘topic modelling’ the order of events was reversed. Topic models grew out of a desire to model collections of text documents, clustering documents into groups that are on a common topic, while allowing that each document might span more than one topic. So, just as with admixture models, each document i is characterized by a vector of topic memberships, $q_{i}$, which is to be estimated. Methods for obtaining point estimates by maximum likelihood were published in [39,40], with Bayesian methods only subsequently introduced in [41] (based on variational approximation) and [42] (based on MCMC). My understanding is that, in contrast to admixture models, Bayesian methods for fitting topic models remain more widely used in practice than maximum-likelihood methods. Nonetheless, maximum-likelihood methods may have computational advantages in this setting too [43].

### (b) Genotype imputation

My second example comes from genetic association studies, which aim to assess the association between genetic variants and an outcome of interest, which here I assume to be a quantitative variable (e.g. height).

A typical genetic association study will consider millions of genetic variants, each of which can be considered to be a binary variable with two possible types (alleles). Because each individual carries two copies of their genome (one from each parent) they may possess either zero, one or two copies of each allele at each genetic variant. Thus, the genotype $x_{ij}$ for an individual i at a genetic variant j is usually reported as the number of copies of one of the alleles chosen as a reference allele, $x_{ij}\in \{0,1,2\}$. Genetic association analysis aims to learn about the relationship between these genotypes and the outcome variable y measured on the same set of individuals. For example, one way to do this is to fit the multiple linear regression model (2.14) and attempt to learn the parameters β.

If both y and X are completely observed then there are many ways to fit such a regression model, including the ridge regression and lasso mentioned above, as well as Bayesian methods [44]. However, for reasons that need not concern us here, it is often the case that some $x_{ij}$ are not directly measured. Instead, we observe data D that inform us about the values of X. For simplicity here, assume that we can sample from the conditional distribution p(X|D) and we can compute the expectation, $\bar{X}:=E(X|D)$, which provides a natural ‘point estimate’ for the genotype matrix X from the data D.

Being a trained Bayesian, I know how to attack this problem: specify *a prior* distribution for β, and compute the posterior distribution for β given the observed data, D and y. Under some simple but reasonable assumptions^6^ the posterior distribution is given by

3.1 p(β|D,y)=∫p(β|X,y)p(X|D) dX,

which, since we can sample from p(X|D), can be approximated by

3.2 $$p(\beta |D,y)\approx \left(\frac{1}{M}\right)\sum_{m=1}^{M}p(\beta |X^{m},y),$$

where $X^{1},\ldots ,X^{M}$ are samples from p(X|D). This is, in essence, the approach I took to this problem, with Servin, in [45]. (If $p(\beta |X^{m},y)$ is not analytically available then this adds an additional layer of complexity that may be dealt with in various ways, usually involving Markov Chain Monte Carlo methods.)

However, there is a simpler way that appeals, intuitively, to many analysts: simply replace, or ‘impute’ the unobserved X with its expectation $\bar{X}$, and then proceed with analysis as X were fully observed. That is, compute $p(\beta |\bar{X},y)$. I will call this the ‘imputation-based’ approach. This approach, which ignores uncertainty in the imputed $\bar{X}$, is anathema to a Bayesian. And yet in essence it has become the approach of choice in this field, and it is an approach that I myself now routinely use.

Some of the computational benefits of this imputation-based approach should be obvious. For example, computing (3.2) takes approximately M times longer than computing $p(\beta |\bar{X},y)$. Since M could be large this could be a non-trivial issue. Maybe more important than computational time is the issue of storage: X is often large, involving millions of variants in tens or hundreds of thousands of individuals, so storing M imputations is unappealing. As a result, when using estimators such as (3.2) the sampling would have to be redone for every new analysis. By contrast, the imputation of $\bar{X}$ can be done once, and then re-used for as many analyses as one likes. This issue becomes particularly salient when data are to be shared across large numbers of analysts. For example, at the time of writing, the UK Biobank [46] distributes $\bar{X}$ for approximately 90 million imputed variants in 500 k individuals to (I am guessing) thousands of authorized users. Because the imputation is handled by the data provider this saves the individual labs who use these data from learning, installing or running any of the imputation infrastructure (i.e. the data D and software required to compute p(X|D)).

Perhaps more surprisingly, the statistical downside of using $p(\beta |\bar{X},y)$ instead of a more rigorous Bayesian approach is not so great as one might expect. Indeed, in [19], we found that $p(\beta |\bar{X},y)$ could, in practice, produce more reliable analysis results than the Monte Carlo approximation (3.2) even for quite large M. In essence this is because, in high dimensions, the variance of the Monte Carlo estimate (3.2) can be too high to ensure reliable inferences β in all directions. That is, while $p(\beta |\bar{X},y)$ is a rather coarse approximation to the posterior, its deterministic nature avoids the problems caused by variance in Monte Carlo approximations. Arguments for deterministic Bayesian approximations over their Monte Carlo counterparts have also been given elsewhere in other settings; see [47], for example.

Finally, for this example, I want to draw attention to what I think is a key attractive feature of the imputation approach, which is its *modularity*. That is, the imputation approach splits the problem into simple parts—(i) compute E(X|D) and (ii) fit (2.14)—which can be studied and optimized separately. If someone comes up with a new improved way of doing (ii) (or wants to embroider the model in (ii) to allow for additional features of the problem) then the imputer can easily use it. On the other hand, if someone finds a better way to do the imputation (i.e. a better way to compute E(X|D)) then this is also easily accommodated within the analysis pipeline. Modularity is a key and explicit feature of the way that computer scientists design software; and mathematicians also work in a modular way when they state lemmas, propositions and theorems. Arranging statistical analysis in a modular way has similar—but perhaps underappreciated—benefits; see [48,49] for another example of this and further discussion. Unfortunately, modularity sometimes involves compromising on the desire for Bayesian methods to propagate uncertainty through all stages of an analysis, but I believe that this is a compromise that is sometimes worth making.

### (c) Discussion

My examples of Bayesian blinkers share something in common: they are examples where point estimates are, in essence, enough. While statisticians in general, and Bayesians in particular, are rightly concerned with uncertainty, it seems worth recognizing that in some settings a good point estimate is enough. Indeed, on the Internet, examples like this abound. When Google displays a ranked list of hits from an Internet search, it does so without any indication of uncertainty in its ranking. When Netflix or Amazon recommends you a movie or book, it provides, at best, a point estimate of its assessment that you will like it. Away from the Internet, few papers that show results from a clustering or a principal components analysis report uncertainties in their results, and while in some cases this might be problematic, it is not always so.

More generally, the lesson I have personally drawn from experience is that it is sometimes worth compromising on statistical principle (e.g. propagating uncertainty) to simplify computation. I see EB methods [11,50,51] as a useful example of this compromise.

I end by acknowledging that discussions of the connections and differences between Bayesian and non-Bayesian methods abound. I have cited some relevant papers along the way, but there are many more, including [52–56] just to start a list.

## Data Availability

This article has no additional data.

## Footnotes

1 Savage’s example of weighing a potato was presumably chosen, in part, because it falls outside this setting.

2 To be more specific about the problems this will cause: although on average 95% of (95%-) confidence intervals will cover the true parameter value, those intervals that include 0 will have a higher than claimed coverage, and those intervals that exclude 0—which are the intervals that analysts tend to focus on—will have a lower than claimed coverage.

3 Perhaps the question itself might seem startling to readers used to accepting p-values as ‘the’ way of measuring evidence against a null hypothesis; however, among Bayesian statisticians, p-values are far from universally regarded as a sensible measure of evidence, and as the analysis here shows, ranking hypotheses by p-values involves making non-trivial implicit assumptions.

4 Interestingly, they seem to think that this viewpoint may scare some readers away, and add ‘For those that do not like the philosophical implications of assuming β to be a random variable, all this is equivalent to constrained estimation by a nonuniform weighting on the values of β.’ (whatever that might mean!).

5 Here, I deliberately use the descriptor ‘non-Bayesian’ rather than ‘frequentist’ and do not consider them synonymous. For example, both maximum-likelihood estimation and penalized approaches like the lasso are non-Bayesian, but, in contrast to confidence intervals and p-values, not inherently frequentist. Of course, one may study the frequency properties of these procedures, but one may also study the frequency properties of Bayesian methods.

6 Specifically, assuming p(β|X,y,D)=p(β|X,y) and p(X|D,y)=p(X|D).
